# Prevalence, Awareness, and Sociodemographic Determinants of Disc Herniation Among Adults in Saudi Arabia

**DOI:** 10.3390/healthcare14101309

**Published:** 2026-05-12

**Authors:** Yahya H. Khormi, Mohammad A. Jareebi, Afrah M. Humadi, Saja A. Almraysi, Ali Y. Madkhali, Saja S. Alqahtani, Eyad M. Albarrati, Abdulaziz M. Alibrahim, Saud N. Alwadani, Ahlam A. Harthi, Weam S. Alqattan, Roaa A. Bajafar, Najla A. Alhazmi, Ibrahim A. Hakami, Farjah H. Algahtani

**Affiliations:** 1 Neurosurgery, Department of Surgery, Faculty of Medicine, Jazan University, Jazan 45142, Saudi Arabia; khormins@gmail.com; 2 Department of Family and Community Medicine, Faculty of Medicine, Jazan University, Jazan 45142, Saudi Arabia; 3 Faculty of Medicine, Jazan University, Jazan 82224, Saudi Arabia; afrahmosa2030@gmail.com (A.M.H.); sajaalasiri1@gmail.com (S.A.A.); ali27yahyamad@gmail.com (A.Y.M.); sajasaeedq@gmail.com (S.S.A.); eyad.albarrati@gmail.com (E.M.A.); abdulaziz1811m@gmail.com (A.M.A.); saudwadani78@gmail.com (S.N.A.); ahlam142323@gmail.com (A.A.H.); najla.alhazmi12@gmail.com (N.A.A.); 4 College of Medicine, Imam Abdulrahman Bin Faisal University, Dammam 34212, Saudi Arabia; weamsalah122@gmail.com; 5 Faculty of Medicine, Umm Al-Qura University, Makkah 24382, Saudi Arabia; ruaabajafar@gmail.com; 6 Orthopedic Surgery, Dawadmi College of Medicine, Shaqra University, Dawadmi 17471, Saudi Arabia; ihakami@su.edu.sa; 7 Oncology Center, Chair of Epidemiology and Public Health Research, Faculty of Medicine, King Saud University/King Saud Medical City, Riyadh 12373, Saudi Arabia; falgahtani@ksu.edu.sa

**Keywords:** disc herniation, public awareness, prevalence, Saudi Arabia, risk factors, sociodemographic determinants, cross-sectional study, logistic regression

## Abstract

**Background/Objectives**: Disc herniation, also termed herniated nucleus pulposus (HNP), is a common spinal disorder affecting approximately 10% of the global population, associated with pain, neurological complications, and diminished quality of life. Despite its global burden, regional variations in public awareness and sociodemographic determinants remain inadequately characterized, particularly in Middle Eastern populations. This study aimed to assess the prevalence, public awareness, and sociodemographic determinants of HNP among adults in Saudi Arabia at a nationwide level. **Methods**: An analytical cross-sectional study was conducted from December 2024 to July 2025. Using a convenience sampling approach via social media platforms, an online questionnaire was distributed nationwide across Saudi Arabia. Data from 1112 participants were analyzed using descriptive statistics and multiple logistic regression. The questionnaire comprised two sections: sociodemographic characteristics and knowledge and awareness of HNP. **Results**: The prevalence of disc herniation was 8.9%, consistent with global estimates. Overall awareness was relatively high at 67.6%, though knowledge of specific risk factors varied considerably. Most participants recognized obesity (88.0%), poor sitting posture (85.8%), history of lower back trauma (86.2%), and work requiring physical effort (88.8%) as risk factors, while fewer acknowledged smoking (46.4%), diabetes (51.2%), sleeping on a soft bed (36.9%), and increased height (35.9%). Multiple logistic regression, adjusted for all sociodemographic, lifestyle, and health-related covariates, identified significant independent predictors of HNP including marital status (married OR = 2.90), current smoking (OR = 2.91), hyperlipidemia (OR = 1.86), family history (OR = 8.95), and prior knowledge of the condition (OR = 2.28). Knowledge of HNP was significantly associated with university education (OR = 1.70), higher income levels (OR = 2.23 for ≥15,000 SAR; OR = 2.07 for 5000–9999 SAR), and family history (OR = 4.70), while those in low and medium workload jobs demonstrated lower knowledge. **Conclusions**: Although overall public awareness of HNP is relatively high in Saudi Arabia, substantial gaps persist in knowledge of modifiable risk factors, particularly smoking and diabetes mellitus. Targeted smoking cessation campaigns, diabetes awareness programs, and ergonomic education initiatives delivered through primary healthcare centers, workplaces, and schools are recommended.

## 1. Introduction

The intervertebral disc comprises the nucleus pulposus, annulus fibrosus, and cartilage endplates, providing spinal flexibility and load distribution. Age-related degeneration or trauma can compromise disc integrity, leading to herniation of nuclear material through the annulus [1,2].

Disc herniation, also termed herniated nucleus pulposus (HNP), represents a common spinal pathology characterized by displacement of nuclear material through a compromised annulus fibrosus, frequently resulting in compression of adjacent neural structures [2]. Clinical manifestations range from localized back pain and radiculopathy to neurological deficits, with severe cases presenting as cauda equina syndrome. HNP represents a leading cause of disability worldwide, imposing substantial economic and healthcare burdens across populations. The lumbar spine, particularly L4–L5 and L5–S1, is most frequently affected due to greater biomechanical stress. While most cases respond to conservative management including NSAIDs and physiotherapy, a subset requires surgical decompression [3].

The etiology of HNP predominantly involves age-related degenerative disc disease, with trauma as the second most common contributing factor [3]. Established risk factors for lumbar HNP include middle age (30–50 years), cigarette smoking, elevated body mass index (BMI), and cardiovascular comorbidities, with particular associations noted among women. Occupational exposures involving repetitive forward flexion, heavy lifting, or manual material handling demonstrate strong associations with herniation due to increased lumbar loading and repetitive strain [3].

In Saudi Arabia, several regional studies have examined HNP awareness. A 2016 study conducted in Taif involving 1034 participants assessed awareness levels regarding HNP, revealing adequate knowledge about the disease entity itself but demonstrating deficiencies in understanding preventive measures and risk factors [4]. A similar investigation conducted in the province of Aseer in March 2019 with 1044 participants reported inadequate awareness across all aspects of HNP [5]. Additionally, a June 2020 study in Jeddah involving 1026 participants measuring awareness levels among the general population and medical students identified poor knowledge regarding preventive measures, disease symptoms, the most commonly affected spinal levels, and the gold standard diagnostic imaging modality [6]. Previous studies conducted in Taif, Aseer, and Jeddah were limited to single cities or provinces, used varying methodology, and lacked nationally representative samples. Critically, none provided a comprehensive nationwide assessment integrating both prevalence estimates and sociodemographic determinants simultaneously. Unlike prior regional investigations, this nationwide study uniquely combines prevalence estimation, awareness assessment, and multivariable analysis of sociodemographic determinants, providing a comprehensive evidence base to inform national public health policy in Saudi Arabia.

## 2. Materials and Methods

### 2.1. Study Design and Setting

An analytical cross-sectional study was conducted in the Kingdom of Saudi Arabia between December 2024 and July 2025. The study targeted all residents of Saudi Arabia aged 18 years and above who were able and willing to provide informed consent. Individuals unable or unwilling to provide consent were excluded from participation.

### 2.2. Sampling and Sample Size Calculation

Participants were recruited using a convenience sampling technique through online platforms. This approach was selected due to its practicality, cost-effectiveness, and ability to rapidly reach a geographically dispersed nationwide sample within the available timeframe and resources. While this method does not yield a probability-based sample, it has been widely employed in similar awareness and prevalence studies across the region. Based on the total population aged 18 years and above in Saudi Arabia (22,091,634) according to the latest census [7], and using a 95% confidence level and a 5% margin of error, the minimum required sample size was calculated as 385 participants, according to the following formula:

$$n^{0}=\frac{Z^{2}\cdot p\cdot (1-p))}{e^{2}}$$

where Z denotes the standard score corresponding to the selected confidence level (1.96 for a 95% CI), p indicates the anticipated prevalence or proportion (0.5 for a conservative estimate), and e represents the allowable margin of error. Adjustments to account for a 15% non-response rate and 10% missing data increased the target sample size to 453. Additionally, to maximize statistical precision, improve subgroup analyses across multiple sociodemographic strata, and ensure adequate power for multivariable logistic regression with numerous covariates, the sample size was expanded by an additional 547 participants, bringing the total target to 1000 participants. Ultimately, 1112 responses were collected. It should be noted that convenience sampling via social media platforms inherently favors individuals who are younger, educated, and digitally active, which may limit the representativeness of the sample with respect to older adults, rural residents, and those with lower digital literacy. Therefore, the findings of this study should not be interpreted as fully representative of the general Saudi adult population. Older adults, rural residents, and individuals with lower digital literacy are underrepresented, which may affect the generalizability of both prevalence and awareness estimates.

### 2.3. Data Collection Tool

An online self-administered questionnaire was developed following a comprehensive literature review and distributed through social media platforms, including WhatsApp, Telegram, and X (formerly Twitter). The questionnaire comprised two main sections: (1) sociodemographic and health characteristics, and (2) knowledge and awareness of HNP. An introductory statement explaining the purpose of research, objectives, and procedures was provided at the beginning of the questionnaire. Electronic informed consent was obtained from all participants before they proceeded to complete the survey. Prior to full deployment, the questionnaire was pilot tested on 30 participants to assess clarity, comprehension, and completion time. Internal consistency of the 11-item knowledge and awareness scale was evaluated on the pilot sample (n = 30) using Cronbach’s alpha, yielding α = 0.78, which indicates acceptable internal consistency for a brief health-knowledge instrument. Expert content validity was established through review by specialists in neurosurgery, orthopedics, and public health, and necessary modifications were made prior to final distribution.

#### 2.3.1. Section 1: Sociodemographic and Health Characteristics

The sociodemographic section collected information on age, gender, weight, height, body mass index (BMI, calculated from self-reported height and weight), nationality, marital status, residence type (city or village), educational attainment, monthly income level, and employment status. Additional health-related information included smoking status (never smoked, former smoker, current smoker) and chronic medical conditions (diabetes mellitus, hypertension, hyperlipidemia, asthma, sickle cell disease, thalassemia, hyperthyroidism, hypothyroidism, rheumatoid arthritis, and hernia). Family history of HNP was also assessed.

#### 2.3.2. Section 2: Knowledge and Awareness of HNP

The second section assessed knowledge and awareness regarding HNP, presented in two parts. The first component evaluated participants’ general awareness of disc herniation through a binary question (yes/no), knowledge of associated risk factors, understanding of protective and preventive measures, and familiarity with diagnostic approaches. It is important to note that this binary question captures basic familiarity with the condition (awareness) rather than in-depth understanding (knowledge) or the ability to apply health information (health literacy). These three constructs are conceptually distinct and should not be used interchangeably.

Participants were asked to identify whether specific factors (advanced age, increased height, smoking, diabetes mellitus, obesity, family history, history of lower back trauma, sleeping on a soft bed, poor sitting posture, prolonged sitting exceeding six hours daily, and physically demanding work) constituted risk factors for disc herniation. The second component specifically targeted participants who had received a formal diagnosis of HNP and included detailed questions about diagnosis timing (less than 3 months, 1–3 months, 3–6 months, 6 months–1 year, more than 1 year), treatment modalities received (surgical versus non-surgical), and symptom duration.

It should be noted that HNP diagnosis was based entirely on self-report, with no clinical verification or diagnostic validation question included in the questionnaire.

### 2.4. Statistical Analysis

Following data collection, the raw dataset was transferred to Microsoft Excel (Microsoft Corporation, Redmond, WA, USA) for preliminary data cleaning, error detection, and validation. Subsequent statistical analyses were performed using R software (version 4.2.3, R Foundation for Statistical Computing, Vienna, Austria). Continuous variables were presented as means ± standard deviations (SD), while categorical variables were presented as frequencies and percentages.

Multiple logistic regression analyses were conducted to identify independent predictors of two primary outcomes: (1) diagnosis of HNP, and (2) knowledge of HNP. All variables were entered simultaneously into the model based on prior literature and theoretical relevance, without data-driven selection procedures. This forced-entry approach ensures adjustment for all plausible confounders regardless of statistical significance. Multicollinearity was assessed using Variance Inflation Factors (VIF); all values were below 5.0, confirming no problematic collinearity among predictors. Categorical variables with more than two categories, such as education, income, marital status, smoking status, and work sector, were coded using treatment/dummy coding with predefined reference categories. Model fit was evaluated using the Hosmer–Lemeshow goodness-of-fit test and the Area Under the Receiver Operating Characteristic Curve (AUC), with AUC > 0.70 considered acceptable and >0.80 considered good. Odds ratios (OR) with 95% confidence intervals (CI) were calculated to quantify the strength and precision of associations. Statistical significance was set at *p* < 0.05.

### 2.5. Ethical Considerations

This study received ethical clearance from the Standing Committee for Scientific Research at Jazan University (Reference No. REC-46/07/1341, dated 9 January 2025). Prior to participation, all individuals were informed about the study objectives, potential outcomes, and their rights, including voluntary participation and confidentiality protections. Electronic informed consent was obtained from all participants before accessing the online questionnaire. The study adhered to the ethical principles outlined in the Declaration of Helsinki and followed the Strengthening the Reporting of Observational Studies in Epidemiology (STROBE) guidelines to ensure methodological rigor and transparent reporting of this cross-sectional investigation.

### 2.6. Use of Generative Artificial Intelligence

A generative AI tool (ChatGPT-5 (OpenAI)) was used solely for language editing. The study design, literature synthesis, data extraction, analysis, and interpretation were entirely conducted by the authors.

## 3. Results

### 3.1. Sociodemographic Characteristics of Participants

Of 1112 participants, the sample predominantly comprised female (65.2%), Saudi national (97.7%), single (62.9%), and university educated (75.0%), with a mean age of 30.7 ± 11.6 years (Table 1).

### 3.2. Health-Related and Habitual Characteristics

The majority of participants had never smoked (84.7%), and prevalence of chronic conditions was generally low, with hyperlipidemia (10.7%) and asthma (9.3%) being the most common. Hematological disorders were uncommon, with sickle cell disease present in 3.1% (n = 35) and thalassemia in 1.6% (n = 18). Thyroid disorders were reported by 7.6% of participants, including hypothyroidism (5.7%, n = 63) and hyperthyroidism (1.9%, n = 21). Rheumatoid arthritis was present in 4.0% (n = 44), and 2.2% (n = 25) reported a history of hernia (Table 2).

### 3.3. HNP Awareness and Prevalence

Participants demonstrated relatively high awareness of HNP, with 67.6% (n = 752) reporting familiarity with the condition. Among all participants, 47.2% (n = 525) indicated knowledge of risk factors associated with HNP, and 24.6% (n = 274) reported a positive family history. The prevalence of self-reported, previously diagnosed HNP was 8.9% (n = 99). Among those diagnosed, the majority (65.7%, n = 65) received their diagnosis more than one year prior to study participation, while 13.1% (n = 13) were diagnosed less than three months ago, 10.1% (n = 10) between six months and one year, and 9.1% (n = 9) between three and six months. Two participants (2.0%) reported diagnosis within one to three months. Regarding treatment modalities, 87.9% (n = 87) of diagnosed patients received non-surgical treatment (medications or physiotherapy), while 12.1% (n = 12) underwent surgical intervention (Table 3).

### 3.4. Knowledge of HNP Risk Factors

**Figure 1:**
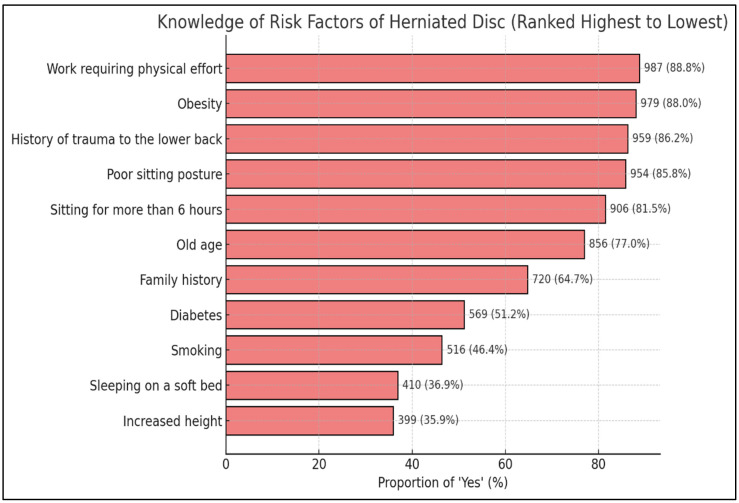
Knowledge of risk factors for HNP among study participants, ranked from highest to lowest proportion of recognition (N = 1112). Bar chart showing percentages in descending order: Physical effort work (88.8%), Obesity (88.0%), Lower back trauma (86.2%), Poor sitting posture (85.8%), Prolonged sitting (81.5%), Advanced age (77.0%), Family history (64.7%), Diabetes (51.2%), Smoking (46.4%), Soft bed (36.9%), Increased height (35.9%).

Participants’ knowledge of specific risk factors for HNP varied considerably. The most widely recognized risk factors included work requiring physical effort such as frequent lifting, bending, or twisting (88.8%, n = 987), obesity (88.0%, n = 979), history of lower back trauma (86.2%, n = 959), and poor sitting posture (85.8%, n = 954). Additionally, 81.5% (n = 906) identified prolonged sitting exceeding six hours daily as a risk factor. Advanced age (77.0%, n = 856) and positive family history (64.7%, n = 720) were also frequently recognized. Conversely, less commonly identified risk factors included diabetes mellitus (51.2%, n = 569), smoking (46.4%, n = 516), sleeping on a soft bed (36.9%, n = 410), and increased height (35.9%, n = 399). These findings demonstrate variable knowledge levels across different risk factors (Table 4, Figure 1).

### 3.5. Determinants of HNP: Multiple Logistic Regression Analysis

Multiple logistic regression analysis identified several significant predictors of HNP diagnosis. Marital status emerged as a strong predictor, with divorced or widowed individuals demonstrating substantially elevated odds (OR = 12.49, 95% CI: 3.65–41.84, *p* < 0.001) and married participants showing increased odds (OR = 2.90, 95% CI: 1.35–6.33, *p* = 0.007) compared to single individuals. Current smoking was significantly associated with HNP (OR = 2.91, 95% CI: 1.34–6.13, *p* = 0.006), while former smoking showed no significant association (OR = 0.96, 95% CI: 0.35–2.40, *p* = 0.939). Hyperlipidemia demonstrated a significant association (OR = 1.86, 95% CI: 1.05–3.30, *p* = 0.034). The strongest predictor was positive family history of HNP (OR = 8.95, 95% CI: 5.34–15.43, *p* < 0.001). Prior knowledge of HNP was also significantly associated with diagnosed disease (OR = 2.28, 95% CI: 1.11–4.25, *p* = 0.001). Other variables including age, sex, BMI, residence, education level, work sector, income, diabetes mellitus, and hypertension did not show significant independent associations with HNP (Table 5).

### 3.6. Determinants of HNP Knowledge: Multiple Logistic Regression Analysis

Multiple logistic regression analysis identified several significant predictors of HNP knowledge among participants. University-level education was significantly associated with greater knowledge (OR = 1.70, 95% CI: 1.20–2.41, *p* = 0.003) compared to secondary education or less, while postgraduate education showed no significant association (OR = 1.61, 95% CI: 0.75–3.57, *p* = 0.225). Higher monthly income was strongly associated with increased knowledge, with participants earning ≥15,000 SAR (OR = 2.23, 95% CI: 1.53–3.27, *p* < 0.001) and those earning 5000–9999 SAR (OR = 2.07, 95% CI: 1.36–3.19, *p* = 0.001) demonstrating significantly greater knowledge compared to those earning <5000 SAR. The strongest predictor of knowledge was positive family history of HNP (OR = 4.70, 95% CI: 3.16–7.21, *p* < 0.001). Interestingly, participants employed in low workload jobs (OR = 0.40, 95% CI: 0.18–0.95, *p* = 0.034) and medium workload jobs (OR = 0.51, 95% CI: 0.31–0.82, *p* = 0.006) demonstrated significantly lower knowledge compared to unemployed individuals. Other variables including age, sex, BMI, residence, marital status, smoking status, and chronic medical conditions did not show significant independent associations with HNP knowledge (Table 6).

## 4. Discussion

### 4.1. Prevalence of HNP

This study revealed a HNP prevalence of 8.9% among adult residents of Saudi Arabia, based on self-reported previous diagnosis. This prevalence estimate should be interpreted in light of its reliance on self-reported diagnosis, which may not reflect clinically confirmed cases. In addition, the convenience-sampling frame, dominated by younger, urban, and university-educated participants, plausibly biases the observed 8.9% downward relative to the true population prevalence, since older and rural adults in whom HNP is more common are underrepresented. This prevalence aligns closely with the approximately 10% global prevalence reported in the literature [8]. However, considerable variation exists across different populations and geographic regions. A study of residents aged 18 years or older in Gansu, China, reported a substantially higher prevalence of 22.77% for lumbar HNP [9]. Within Saudi Arabia, previous studies have documented varying prevalence rates. The current finding of 8.9% is lower than the 15% prevalence of disc bulge reported in Taif city [10]. The lower prevalence observed may reflect differences in study methodology, diagnostic criteria, and healthcare-seeking behaviors, rather than a true difference in disease burden. The consistency with global estimates suggests that HNP represents a significant health concern in Saudi Arabia, warranting attention from public health authorities and healthcare providers.

### 4.2. Awareness and Knowledge Patterns

Participants in this study demonstrated a considerable level of awareness regarding HNP, with 67.6% reporting familiarity with the condition. This finding suggests that a substantial proportion of the general population possesses basic awareness of HNP as a health condition. However, participants exhibited varying levels of knowledge about specific risk factors. Obesity (88.0%), history of lower back trauma (86.2%), work requiring physical effort (88.8%), and poor sitting posture (85.8%) were widely recognized as significant risk factors. Furthermore, 81.5% of participants identified prolonged sitting exceeding six hours daily as a risk factor. Family history (64.7%) and advanced age (77.0%) were also frequently acknowledged. These findings are consistent with earlier research conducted in Taif, which similarly reported good awareness of certain risk factors among the public [4]. In contrast, fewer participants recognized diabetes mellitus (51.2%), smoking (46.4%), sleeping on a soft bed (36.9%), and increased height (35.9%) as risk factors. Sleeping on a soft mattress has been proposed to reduce lumbar spinal support during rest and may, in some studies, contribute to greater disc loading; however, the evidence remains inconsistent and a soft sleeping surface is best considered a debated rather than established risk factor for HNP [11].

Increased standing height has likewise been suggested to impose greater mechanical stress on intervertebral discs through longer spinal lever arms, but the association with clinically diagnosed HNP is indirect and not consistently demonstrated across populations [12]. These knowledge gaps are clinically significant, as smoking accelerates disc degeneration through impaired vascular supply and oxidative stress, while diabetes mellitus promotes disc degeneration via advanced glycation end-products and microvascular compromise. Poor recognition of these modifiable risk factors represents a missed opportunity for prevention and warrants targeted public health education. The high overall awareness level contrasts with research conducted in Jeddah, which found that only 54.1% of the general population understood the condition well [6]. This discrepancy may reflect temporal trends toward improved health literacy, differences in study populations, or variations in data collection methodologies. The relatively good awareness identified in our study may be attributed to increased access to health information through digital media, health education campaigns, or personal or family experiences with the condition.

### 4.3. Sociodemographic and Lifestyle Determinants of HNP

Multiple significant determinants emerged. Marital status strongly predicted HNP, with divorced/widowed individuals showing markedly elevated odds, resembling previous Saudi studies on back pain [5,13]. The mechanisms underlying this association remain unclear and warrant further investigation in longitudinal studies. Possible explanations for example, age-related accumulation of risk among ever-married individuals, or psychosocial stress and reduced social support among the divorced/widowed are offered here strictly as hypotheses to be tested in adequately powered, longitudinal studies, and should not be interpreted as established mechanisms. The very small divorced/widowed subgroup (n = 29) and the correspondingly wide 95% confidence interval for the adjusted odds ratio (3.65–41.84) make this estimate statistically unstable. The point estimate of 12.49 should therefore not be read as a precise effect size; rather, it indicates a direction and magnitude that is highly uncertain and that could shift substantially with even a small change in the number of cases. These associations, therefore, point toward areas deserving further investigation rather than definitive conclusions. Longitudinal studies with larger and more representative samples would be a natural next step to clarify the directionality and strength of these relationships. This finding is consistent with previously reported associations between marital status and musculoskeletal disorders in the literature, though the contributing factors remain to be fully characterized. Current smoking was independently associated with self-reported HNP in the adjusted model. This finding is compatible with prior literature suggesting that nicotine may impair disc nutrition and promote oxidative stress and cellular injury [13,14]. Prospective longitudinal studies are needed to confirm whether smoking represents a true independent risk factor for disc herniation in this population. The association between hyperlipidaemia and HNP is consistent with the hypothesis that systemic metabolic and inflammatory pathways may influence disc health [15]; however, given the cross-sectional design, reverse causation and residual confounding cannot be excluded.

Family history emerged as the strongest predictor, consistent with international and local research highlighting genetic contributions to disc herniation [5,14]. Prior knowledge of HNP was associated with a higher rate of self-reported diagnosis. This is most plausibly bidirectional, having a diagnosis prompts learning about the condition, and pre-existing awareness may increase healthcare-seeking and the likelihood of receiving a formal diagnosis and should not be read as evidence that knowledge causes HNP. These findings demonstrate that HNP results from complex social, behavioral, metabolic, and genetic interactions, necessitating comprehensive prevention strategies targeting smoking cessation, metabolic optimization, and family-based screening.

### 4.4. Determinants of HNP Knowledge

Sociodemographic factors significantly shaped knowledge. University education and higher income (≥15,000 SAR and 5000–9999 SAR) predicted greater self-reported knowledge of HNP, consistent with the literature documenting education and socioeconomic status as predictors of health-related knowledge [5,16]. Because our instrument captured factual familiarity rather than the ability to apply or appraise health information, we frame this as a difference in disease-specific knowledge and not as a measure of health literacy in the formal sense. Family history strongly influenced knowledge, reinforcing the findings from Aseer in which affected relatives were associated with increased awareness [5], highlighting opportunities for family-centered education interventions.

The interpretations that follow are advanced as hypotheses generated by our cross-sectional data rather than as established explanations. Counterintuitively, participants employed in low and medium workload jobs demonstrated significantly lower knowledge of HNP compared to unemployed individuals. This finding may reflect several plausible mechanisms. Unemployed individuals may have more discretionary time for health information-seeking, greater engagement with digital media and social platforms, or may include students and homemakers with ongoing exposure to educational content [17]. In contrast, workers in low- and medium-workload occupations, such as clerical, administrative, or light service roles, may experience moderate time constraints without the compensatory occupational health exposure associated with high-demand physical jobs, where ergonomic training and musculoskeletal awareness are more routinely provided [18]. Additionally, these workers may perceive themselves as insufficiently at-risk to seek out condition-specific health information, a phenomenon consistent with the precaution adoption process model, in which individuals who do not perceive personal vulnerability remain in earlier stages of health awareness [19]. These interpretations remain speculative, however, and longitudinal studies with objective occupational classification and a validated health-knowledge or health-literacy instrument (e.g., the HLS-EU-Q or a disease-specific equivalent) are needed to clarify the directionality and underlying drivers of this association [5].

### 4.5. Public Health Implications

Within the limits of an observational, self-report design, our findings suggest that public-health initiatives could usefully address smoking, metabolic health, ergonomic principles, and physical-activity patterns, while recognizing that the present data do not by themselves establish the effectiveness of any specific intervention. The identified gaps in knowledge regarding specific risk factors, particularly modifiable factors such as smoking, indicate opportunities for targeted health education campaigns. Public health initiatives should emphasize the role of smoking cessation in preventing disc degeneration, promote awareness of metabolic health optimization, educate the public about ergonomic principles and proper posture, and encourage appropriate physical activity patterns. Such campaigns should target specific high-impact settings including primary healthcare centers for early screening and risk counseling; workplaces, particularly those involving physical labor, for ergonomic training and occupational health programs; schools and universities for early health literacy education among young adults; and community-based platforms for reaching lower-income and rural populations with limited digital access.

The strong associations between family history and both HNP prevalence and knowledge suggest potential value in family-centered screening and education programs. Individuals with a positive family history may represent a higher-risk group that could be considered for earlier risk counselling and structured preventive education in primary-care settings; the appropriateness and yield of any such targeted approach would need to be evaluated in dedicated studies. Healthcare providers should routinely assess family history of HNP and provide personalized risk counseling for at-risk individuals.

The socioeconomic gradients observed in both HNP prevalence and knowledge underscore the need for interventions that address health equity. Programs should be designed to reach underserved populations, reduce barriers to healthcare access, provide culturally appropriate and accessible health information, and address social determinants of health that influence both disease risk and health literacy. Equity-oriented programs may particularly benefit individuals with lower educational attainment or income and those in occupational categories under-served by existing occupational-health initiatives, although the optimal design and reach of such programs will require formal evaluation.

### 4.6. Strengths and Limitations

This study’s strengths include a large sample (N = 1112) substantially exceeding minimum requirements, nationwide recruitment capturing diverse geographic regions, analytical cross-sectional design enabling multivariable associations, and comprehensive assessment of sociodemographic, lifestyle, and health factors. Despite these strengths, three limitations must be acknowledged. First, the convenience-sampling design over-represents young, urban, and university-educated participants, so the sample is not nationally representative; consequently, the 67.6% awareness figure is likely an overestimate, and the 8.9% self-reported prevalence is likely an underestimate, of the corresponding population values. Second, HNP status was based entirely on participants’ self-report of a prior diagnosis, with no clinical or radiological verification, which introduces misclassification bias of unknown direction. Third, although the knowledge-and-awareness items were pilot-tested, reviewed by content experts, and showed acceptable internal consistency (Cronbach’s α = 0.78), the instrument has not been validated against a gold-standard health-knowledge or health-literacy measure; awareness in particular was captured by a single binary item, which may overestimate true comprehension. Recall bias inherent to self-administered questionnaires may also have affected responses on lifestyle and comorbidity items.

Future research should prioritize probability-based sampling frameworks, including stratified random sampling and household-level surveys, with deliberate oversampling of rural, elderly, and lower-income populations, alongside clinically verified diagnoses and validated health literacy measures.

## 5. Conclusions

This nationwide cross-sectional study of 1112 adults found an HNP prevalence of 8.9%, consistent with global estimates. Despite relatively high overall awareness (67.6%), substantial gaps persist in knowledge of modifiable risk factors, particularly smoking and diabetes mellitus. Marital status, smoking, hyperlipidemia, and family history were significant independent determinants of HNP, while education and income were key predictors of knowledge, underscoring persistent disease-related knowledge disparities that require equity-focused interventions. However, due to the cross-sectional nature of the study, the findings cannot establish causation. Future studies should employ probability-based sampling frameworks alongside longitudinal study designs to better elucidate causal relationships.

## Figures and Tables

**Table 1:** Sociodemographic characteristics of study participants (N = 1112).

Characteristic	Value
**Continuous variables**	**Mean ± SD**
Age (years)	30.7 ± 11.6
Weight (kg)	65 ± 19
Height (cm)	162 ± 9
BMI (kg/m^2^)	25 ± 6.3
**Categorical variables**	**n (%)**
**Sex**	
Female	725 (65.2)
Male	387 (34.8)
**Nationality**	
Saudi	1086 (97.7)
Non-Saudi	26 (2.3)
**Marital status**	
Single	699 (62.9)
Married	384 (34.5)
Divorced/Widowed	29 (2.6)
**Residence**	
City	816 (73.4)
Village	296 (26.6)
**Education**	
Secondary or less	230 (20.7)
University	834 (75.0)
Postgraduate (Master’s/Doctorate)	48 (4.3)
**Monthly income (SAR)**	
<5000	307 (27.6)
5000–9999	192 (17.3)
10,000–14,999	274 (24.6)
≥15,000	339 (30.5)

SD: standard deviation; BMI: body mass index; SAR: Saudi Arabian Riyal.

**Table 2:** Health-related and habitual characteristics of study participants (N = 1112).

Characteristic	n (%)
**Smoking status**	
Never smoked	942 (84.7)
Former smoker	70 (6.3)
Current smoker	100 (9.0)
**Diabetes mellitus**	
No	1042 (93.7)
Yes	70 (6.3)
**Hypertension**	
No	1020 (91.7)
Yes	92 (8.3)
**Hyperlipidemia**	
No	993 (89.3)
Yes	119 (10.7)
**Asthma**	
No	1009 (90.7)
Yes	103 (9.3)
**Sickle cell disease**	
No	1077 (96.9)
Yes	35 (3.1)
**Thalassemia**	
No	1094 (98.4)
Yes	18 (1.6)
**Hyperthyroidism**	
No	1091 (98.1)
Yes	21 (1.9)
**Hypothyroidism**	
No	1049 (94.3)
Yes	63 (5.7)
**Rheumatoid arthritis**	
No	1068 (96.0)
Yes	44 (4.0)
**Hernia**	
No	1087 (97.8)
Yes	25 (2.2)

**Table 3:** HNP-related variables among study participants (N = 1112).

Characteristic	n (%)
**Awareness of HNP**	
No	360 (32.4)
Yes	752 (67.6)
**Family history of HNP**	
No	838 (75.4)
Yes	274 (24.6)
**Knowledge of risk factors**	
No	587 (52.8)
Yes	525 (47.2)
**Diagnosed with HNP**	
No	1013 (91.1)
Yes	99 (8.9)
Time since diagnosis *	
Less than 3 months	13 (13.1)
1–3 months	2 (2.0)
3–6 months	9 (9.1)
6 months–1 year	10 (10.1)
More than 1 year	65 (65.7)
Treatment received *	
Non-surgical (medication/physiotherapy)	87 (87.9)
Surgical	12 (12.1)

* Percentages calculated among participants diagnosed with HNP (n = 99).

**Table 4:** Knowledge of risk factors for HNP among study participants (N = 1112).

Risk Factor	Recognized as Risk Factor n (%)	Not Recognized n (%)
Work requiring physical effort (lifting, bending, twisting)	987 (88.8)	125 (11.2)
Obesity	979 (88.0)	133 (12.0)
History of lower back trauma	959 (86.2)	153 (13.8)
Poor sitting posture	954 (85.8)	158 (14.2)
Prolonged sitting (>6 h daily)	906 (81.5)	206 (18.5)
Advanced age	856 (77.0)	256 (23.0)
Positive family history	720 (64.7)	392 (35.3)
Diabetes mellitus	569 (51.2)	543 (48.8)
Smoking	516 (46.4)	596 (53.6)
Sleeping on soft bed	410 (36.9)	702 (63.1)
Increased height	399 (35.9)	713 (64.1)

**Table 5:** Multiple logistic regression analysis: determinants of HNP among study participants (N = 1112).

Predictor	Odds Ratio	95% CI	*p*-Value
**Demographics**			
Age (per year increase)	1.03	0.99–1.06	0.122
**Sex** (reference: Female)			
Male	0.92	0.50–1.64	0.771
BMI (per kg/m^2^ increase)	1.00	0.95–1.04	1.000
**Residence** (reference: City)			
Village	1.55	0.87–2.74	0.130
**Socioeconomic**			
**Education** (reference: Secondary or less)			
University	1.35	0.67–2.88	0.421
Postgraduate (Master’s/Doctorate)	0.89	0.25–2.94	0.853
**Marital status** (reference: Single)			
Married	2.90	1.35–6.33	**0.007**
Divorced/Widowed	12.49	3.65–41.84	**<0.001**
**Occupational**			
**Work sector** (reference: No job)			
High workload jobs	1.32	0.29–5.13	0.706
Medium workload jobs	1.22	0.61–2.44	0.569
Low workload jobs	1.38	0.39–4.51	0.606
**Monthly income (SAR)** (reference: <5000)			
5000–9999	0.84	0.36–1.92	0.688
10,000–14,999	0.63	0.29–1.39	0.257
≥15,000	0.56	0.25–1.23	0.151
**Lifestyle**			
**Smoking status** (reference: Never smoked)			
Current smoker	2.91	1.34–6.13	**0.006**
Former smoker	0.96	0.35–2.40	0.939
**Comorbidities**			
**Diabetes mellitus** (reference: No)			
Yes	1.93	0.86–4.20	0.104
**Hypertension** (reference: No)			
Yes	0.76	0.35–1.59	0.471
**Hyperlipidemia** (reference: No)			
Yes	1.86	1.05–3.30	**0.034**
**Genetic**			
**Family history of** HNP (reference: No)			
Yes	8.95	5.34–15.43	**<0.001**
**Prior knowledge of** HNP (reference: No)			
Yes	2.28	1.11–4.25	**0.001**

CI: confidence interval; BMI: body mass index; SAR: Saudi Arabian Riyal. Bold values indicate statistical significance (*p* < 0.05).

**Table 6:** Multiple logistic regression analysis: determinants of HNP knowledge among study participants (N = 1112).

Predictor	Odds Ratio	95% CI	*p*-Value
**Demographic**			
Age (per year increase)	1.02	0.99–1.04	0.153
**Sex** (reference: Female)			
Male	0.93	0.69–1.27	0.667
BMI (per kg/m^2^ increase)	1.02	0.99–1.05	0.183
**Residence** (reference: City)			
Village	1.22	0.90–1.67	0.205
**Socioeconomic**			
**Education** (reference: Secondary or less)			
University	1.70	1.20–2.41	**0.003**
Postgraduate (Master’s/Doctorate)	1.61	0.75–3.57	0.225
**Marital status** (reference: Single)			
Married	1.34	0.86–2.08	0.195
Divorced/Widowed	1.09	0.43–2.97	0.856
**Occupational**			
**Work sector** (reference: No job)			
High workload jobs	0.91	0.35–2.55	0.843
Medium workload jobs	0.51	0.31–0.82	**0.006**
Low workload jobs	0.40	0.18–0.95	**0.034**
**Monthly income (SAR)** (reference: <5000)			
5000–9999	2.07	1.36–3.19	**0.001**
10,000–14,999	1.17	0.79–1.72	0.432
≥15,000	2.23	1.53–3.27	**<0.001**
**Lifestyle**			
**Smoking status** (reference: Never smoked)			
Current smoker	1.38	0.85–2.29	0.207
Former smoker	1.17	0.61–2.35	0.636
**Comorbidities**			
**Diabetes mellitus** (reference: No)			
Yes	0.66	0.34–1.29	0.212
**Hypertension** (reference: No)			
Yes	1.04	0.54–2.05	0.910
**Hyperlipidemia** (reference: No)			
Yes	1.64	0.94–2.95	0.088
**Genetic factor**			
**Family history of** HNP (reference: No)			
Yes	4.70	3.16–7.21	**<0.001**

Predictor categories are grouped by domain for improved readability. Bold values indicate statistical significance (*p* < 0.05).

## Data Availability

The data presented in this study is available on request from the corresponding author. The data is not publicly available due to ethical restrictions and privacy concerns related to sensitive health information.

## Abbreviations

The following abbreviations are used in this manuscript:

BMI	Body Mass Index
CI	Confidence Interval
HNP	Herniated Nucleus Pulposus
IVD	Intervertebral Disc
LDH	Lumbar Disc Herniation
OR	Odds Ratio
SAR	Saudi Arabian Riyal
